# Associations of self−directed learning ability and critical thinking disposition with internship performance indicators in nursing interns

**DOI:** 10.3389/fpubh.2026.1945090

**Published:** 2026-10-16

**Authors:** Caixia Dai, Yongbao Wei, Yuxuan Liang, Li Huang, Shuang Peng

**Affiliations:** 1 Clinical Nursing Teaching and Research Section, The Second Xiangya Hospital of Central South University, Changsha, Hunan, China; 2 Department of Urology, Fuzhou University Affiliated Provincial Hospital, Fuzhou, China; 3 Guilin Hospital of the Second Xiangya Hospital, Central South University, Guilin, China; 4 Department of Urology, The Second Xiangya Hospital, Central South University, Changsha, Hunan, China

**Keywords:** critical thinking, internship performance, learning ability, nursing interns, self directed learning

## Abstract

We aimed to characterize outcome-specific associations of self-directed learning (SDL) ability and critical thinking (CT) disposition with selected routinely collected internship performance indicators among nursing interns. Eighty interns were included in the study. SDL and CT were measured using validated Chinese instruments during the teaching period. Performance outcomes were routinely recorded. Associations were examined using Pearson correlations and multivariable linear regression adjusted for age, sex, and final surgical nursing course score. Sensitivity analyses included Spearman correlations and a regression model excluding the final surgical nursing course score. The mean age of the eighty interns was 21.36 ± 0.70 years. CT total score was not significantly correlated with theory examination score in the Pearson analysis (*r* = 0.003, *p* = 0.980) and was not independently associated with theory examination score after adjustment (*β* = 0.014, 95% CI − 0.103 to 0.131; *p* = 0.817), while SDL total score was also not independently associated with theory examination score (*β* = −0.110, 95% CI − 0.226 to 0.007; *p* = 0.065). Neither SDL nor CT was independently associated with the comprehensive instructor rating (*p* = 0.469 and *p* = 0.361, respectively). In exploratory domain-level analyses, CT open-mindedness was a nominal positive association with weekly assignment performance (*r* = 0.244, *p* = 0.032; Spearman *ρ* = 0.250, *p* = 0.028), and CT inquisitiveness showed a nominal positive association with case presentation performance (*r* = 0.292, *p* = 0.010; Spearman *ρ* = 0.328, *p* = 0.004). However, no domain-level association remained statistically significant after false-discovery-rate adjustment. These domain-level findings should therefore be considered exploratory and hypothesis-generating rather than confirmatory.

**Clinical trial registration:**

ChiCTR2400085591.

## 1 Introduction

Clinical internships mark a transition from classroom learning to bedside practice, where nursing trainees must plan and prioritize tasks, communicate within teams, and make timely judgments in real clinical workflows (1, 2). As a result, internship performance is shaped not only by knowledge acquisition but also by learners’ ability to regulate their learning and integrate information under time pressure (3, 4).

Within nursing education, two learner attributes are often viewed as central to competency growth: self-directed learning (SDL) and critical thinking disposition. SDL is conceptualized as a process in which the learner takes initiative in diagnosing learning needs, formulating goals, identifying resources, implementing strategies, and evaluating outcomes (5, 6); it has been recognized as a prerequisite for lifelong professional development in rapidly evolving clinical environments (7). Critical thinking disposition, operationalized in the present study using an instrument grounded in the seven-dimension Delphi consensus framework (8), reflects a tendency to appraise evidence, consider alternatives, and reason systematically when faced with uncertainty capabilities that support safe, evidence-informed care (9, 10).

However, evidence linking these attributes to internship achievement remains mixed. Previous studies have reported associations between SDL and clinical competence or professional outcomes (7, 11), although findings may vary across settings and outcome types. Similarly, critical thinking disposition has been examined in both nursing students and newly graduated nurses (9, 10), but its relationship with specific performance outcomes appears to vary according to the assessment context; for example, Fero et al. (12) reported no significant association with paper-based vignette performance but a significant association with high-fidelity simulation performance. Many existing studies examine a single endpoint or rely on unadjusted bivariate analyses, limiting their ability to account for confounders or to distinguish between outcome-specific effects (4, 13). Consequently, it remains uncertain which aspects of SDL and critical thinking disposition correspond to specific performance components in routine clinical teaching systems.

Accordingly, this study aimed to (i) quantify outcome-specific associations of self-directed learning ability and critical thinking disposition with multiple routinely recorded internship performance indicators in a urology nursing rotation, and (ii) examine whether these ability constructs align with commonly used assessment components, thereby informing competency-aligned assessment design in clinical nursing education.

## 2 Materials and methods

### 2.1 Study design and setting

This study employed a single center, secondary observational analytic design using data collected from a structured urology nursing teaching study conducted between April 2022 and March 2024. The internship program followed a standardized rotation curriculum, assessment framework, and supervision structure throughout the study period.

Although the data were derived from an organized teaching program, the present analysis was non − interventional in nature and focused exclusively on examining associations between learner ability constructs and routinely recorded performance indicators. No between−group comparisons of teaching interventions were conducted, and no causal inferences were intended.

The parent study was approved by the Ethics Committee of The Second Xiangya Hospital of Central South University (E2024121), and all participants provided written informed consent before participation.

### 2.2 Participants

All nursing interns who completed the full urology rotation during the study period were eligible for inclusion. Inclusion criteria were: (1) enrollment in an accredited nursing education program; (2) completion of prerequisite theoretical nursing coursework; and (3) completion of the required duration of the urology internship with available assessment records.

Interns were excluded if they were absent from the rotation for more than 2 weeks or had no usable internship performance data. A total of 80 interns met the eligibility criteria and were included in the analysis.

### 2.3 Measures and variables

#### 2.3.1 Self−directed learning ability

Self−directed learning ability was assessed using the Self-Directed Learning Ability Scale for Nursing Students developed and validated by Zhang and Li (14), comprising 30 items across four domains: learning motivation, self−management, collaborative learning, and information literacy. The four domains comprise 8, 11, 5, and 6 items, respectively. Items are rated on a five−point Likert scale, yielding a total score range of 30–150, with higher scores indicating stronger self-directed learning ability. In the present cohort, internal consistency was high (Cronbach’s α = 0.945).

#### 2.3.2 Critical thinking disposition

Critical thinking disposition was assessed using the Critical Thinking Disposition Inventory–Chinese Version (CTDI-CV), adapted and validated for Chinese-speaking populations by Pang et al. (15) from the California Critical Thinking Disposition Inventory (CCTDI) (8). The CTDI-CV consists of 70 items across seven dimensions: truth−seeking, open−mindedness, analyticity, systematicity, confidence in reasoning, inquisitiveness, and cognitive maturity.

Items were scored according to the instrument scoring rules, including appropriate reverse coding. Total scores range from 70 to 420, with higher scores indicating a stronger critical thinking disposition. The CTDI-CV assesses disposition toward critical thinking rather than direct critical-thinking ability or clinical reasoning performance. Internal consistency in this study, Cronbach’s α was 0.692.

#### 2.3.3 Internship performance indicators

Internship performance indicators were obtained from the department’s routine evaluation system and were conceptualized as proxies for different facets of clinical learning performance. Rather than treating internship achievement as a single construct, we predefined a set of complementary indicators that vary in their emphasis on knowledge recall, procedural performance, process engagement, clinical synthesis, and global workplace−based appraisal. All indicators were recorded as part of standard educational practice and were extracted in de − identified form for analysis.

Theory examination score served as a proxy for examination−based knowledge performance. This written assessment, scored on a 0–100 scale, primarily reflects the ability to retrieve and apply foundational theoretical knowledge relevant to urology nursing within a test environment. While it represents an important component of competency development, it may also incorporate test−taking strategies and other factors intrinsic to written examinations.

Practical skills score was used as a proxy for procedural competence. Skills assessments were conducted multiple times during the rotation using routine departmental checklists or criteria, and the value analyzed in this study was the mean score across available skill assessments, thereby reducing the influence of any single assessment occasion and providing a more stable estimate of procedural performance.

Weekly assignment score reflected engagement in process−oriented learning activities. Weekly assignments are typically designed to encourage continuous learning behaviors (e.g., review, reflection, structured completion of tasks) and therefore may capture elements of self−regulation and persistence across the rotation. The analytic indicator was the average weekly assignment score across assigned weeks.

Case presentation score was considered a proxy for clinical synthesis and communication. Case presentations require interns to organize clinical information, prioritize key problems, and communicate reasoning in a structured format. Scores were generated under routine teaching practice using a structured rubric on a 0–10 scale and recorded within the teaching system.

Finally, the comprehensive end−of−rotation instructor evaluation represented a workplace−based global appraisal. This composite rating was assigned at rotation completion based on routine observation and documentation and was intended to summarize overall clinical performance across multiple competency dimensions. In practice, such global evaluations may integrate professional behaviors, communication, and overall impression, and may be influenced by rater perceptions and the opportunity to observe performance.

To maximize comparability, the internship program applied consistent documentation conventions and scoring procedures throughout the study period. Practical skills assessments were scored using routine departmental checklists or criteria, case presentations were scored using a structured departmental rubric, and the comprehensive end-of-rotation evaluation was assigned by instructors on the basis of routine observation and documentation. These routine assessments were therefore treated as indicators of internship performance rather than validated direct measures of specific competencies. Effective sample sizes for each indicator were reported with the corresponding analyses, reflecting routine missingness patterns in educational records.

### 2.4 Data management

Data were independently entered by two investigators and cross−checked for consistency. Discrepancies were resolved by reference to original records. Missing data were limited: the final surgical nursing course score was available for 78 of 80 interns, and theory examination and practical skills scores were available for 77 interns each; the other performance indicators and SDL and CT total scores were available for all 80 interns. No missing values were imputed. Correlation analyses were conducted in the matched analytic samples available across the respective data sources (*n* = 74 for SDL analyses and *n* = 77 for CT analyses), reflecting the matching of questionnaire and performance records across data sources, whereas multivariable regression analyses used complete cases for all variables included in the respective model. Accordingly, effective sample sizes varied across analyses and are reported with the corresponding results.

### 2.5 Statistical analysis

Statistical analyses were conducted in SPSS (version 26.0). Continuous variables are summarized as mean ± SD and categorical variables as counts and percentages. Associations between SDL and critical thinking disposition scores and internship performance indicators were first screened using Pearson correlations; Spearman rank correlations were used as a sensitivity analysis to assess robustness to distributional assumptions. Multivariable linear regression was then used to evaluate the independent associations of SDL and critical thinking disposition with theory examination score and comprehensive end of rotation instructor evaluation, adjusting for age, sex, and final surgical nursing course score. Adjusted partial correlations were additionally calculated for selected associations, controlling for age, sex, and final surgical nursing course score. Analyses involving individual SDL and CT domains were considered exploratory. Four SDL domains and seven CT domains were each examined against five internship performance indicators, resulting in 55 domain-level comparisons. To assess the influence of multiple testing, Benjamini–Hochberg false-discovery-rate (FDR) adjustment was applied to the 55 exploratory Pearson comparisons as a sensitivity analysis; the corresponding 55 Spearman domain-level comparisons were adjusted separately using the same procedure, with *q* < 0.05 used as the FDR significance threshold. Covariate selection was theory-informed and constrained by variables available in the parent dataset rather than based on statistical significance in the present analyses. Age and sex were included as basic demographic characteristics that could be associated with educational performance, while the final surgical nursing course score was included as an available proxy for prior academic performance and a potential confounder. As a sensitivity analysis, the theory-score model was refitted without this covariate using the same complete-case sample. Additional sensitivity analyses included refitting the theory-score model without age and fitting a standardized regression model in which the outcome and continuous predictors were standardized as z-scores, with sex retained as a binary variable. Multicollinearity was assessed using variance inflation factors. Overall model fit was summarized using *R*^2^, adjusted *R*^2^, and the overall *F*-test; Residual normality was assessed using the Shapiro–Wilk test, homoscedasticity using the Breusch–Pagan test, linearity/model specification using the Ramsey RESET test, and influential observations using Cook’s distance; residual independence was additionally assessed using the Durbin–Watson statistic. Regression results are reported as unstandardized *β* with SE and 95% CI, and statistical significance was defined as a two−sided *p* < 0.05.

## 3 Results

### 3.1 Demographics and ability measures

Eighty interns were included. Missing data were limited to the final surgical nursing course score (2/80, 2.5%) and selected performance outcomes, resulting in different effective sample sizes across analyses. A summary of variable availability and missingness is provided in Supplementary Table S1. Mean age was 21.36 ± 0.70 years (*n* = 80) (Table 1), and the sample included 5 males (6.3%) and 75 females (93.8%). The final surgical nursing course score was 88.55 ± 6.32 (*n* = 78). Self−directed learning total score averaged 111.98 ± 17.79 (*n* = 80), with domain means of 31.15 ± 5.50 (motivation), 41.44 ± 7.25 (self−management), 17.98 ± 3.35 (collaboration), and 21.41 ± 3.80 (information literacy). Critical thinking total score was 252.59 ± 22.40 (*n* = 80).

**Table 1:** Descriptive statistics of demographics and ability measures.

Variable	*n*	Mean	SD
Age (years)	80	21.36 (range: 20–23 years)	0.70
Final surgical nursing course score	78	88.55	6.32
Self-directed learning total score	80	111.98	17.79
– Motivation	80	31.15	5.50
– Self-management	80	41.44	7.25
– Collaboration	80	17.98	3.35
– Information literacy	80	21.41	3.80
Critical thinking total score	80	252.59	22.40
– Truth-seeking domain	80	35.76	5.12
– Open-mindedness domain	80	37.80	4.92
– Analytical domain	80	36.46	7.11
– Systematicity domain	80	34.61	5.43
– Confidence in reasoning domain	80	35.18	4.15
– Inquisitiveness domain	80	36.23	5.58
– Cognitive maturity domain	80	36.55	5.03

Values are mean ± SD unless otherwise stated.

### 3.2 Internship performance indicators

Mean theory examination score was 85.26 ± 7.94 (n = 77) (Table 2), practical skills average score was 94.19 ± 0.91 (*n* = 77), weekly assignment average score was 90.29 ± 7.09 (*n* = 80), case score was 7.27 ± 0.55 (*n* = 80), and the comprehensive end-of-rotation evaluation score was 92.48 ± 8.37 (*n* = 80).

**Table 2:** Descriptive statistics of performance indicators.

Performance indicator	*n*	Mean (range)	SD
Theory examination score	77	85.26 (50.00–100.00)	7.94
Practical skills average score	77	94.19 (92.33–97.00)	0.91
Weekly assignment average score	80	90.29 (63.33–99.00)	7.09
Case score	80	7.27 (6.50–9.00)	0.55
Comprehensive end-of-rotation evaluation score	80	92.48 (57.00–100.00)	8.37

Values are mean ± SD. Performance indicators were extracted from the routine internship evaluation system; weekly assignment and practical skills scores represent mean scores across recorded assessments during the rotation.

### 3.3 Correlation analysis

In Pearson correlation analyses, associations differed by outcome type. At the total−score level, self−directed learning showed a weak, non − significant relationship with the comprehensive instructor evaluation (*r* = 0.129, *p* = 0.274) (Table 3) and was not significantly correlated with theory score (*r* = −0.164, *p* = 0.162). Critical thinking total score was not significantly correlated with theory score (*r* = 0.003, *p* = 0.980). Across the 55 exploratory domain-level Pearson comparisons, five associations had unadjusted *p* < 0.05 (Supplementary Table S2), including SDL motivation and information literacy with theory score, CT open-mindedness with weekly assignment performance, and CT confidence in reasoning and inquisitiveness with case presentation performance. CT open-mindedness was positively associated with weekly assignment performance (*r* = 0.244, *p* = 0.032), and CT inquisitiveness was positively associated with case presentation performance (*r* = 0.292, *p* = 0.010); both associations also had unadjusted *p* < 0.05 in the Spearman analyses (*ρ* = 0.250, *p* = 0.028 and ρ = 0.328, *p* = 0.004, respectively) (Table 3; Supplementary Tables S2, S3). However, none of the 55 Pearson domain-level associations remained significant after Benjamini–Hochberg FDR adjustment (all *q* ≥ 0.315). Likewise, none of the 55 corresponding Spearman associations remained significant after FDR adjustment (all *q* ≥ 0.197).

**Table 3:** Key Pearson correlations between ability measures and selected outcomes.

Ability measure	Outcome	*n*	*r*	95% CI	*P*
SDL motivation	Theory score	74	−0.261	−0.462 to −0.035	0.025
CT analytical domain	Theory score	77	0.112	−0.115 to 0.328	0.331
SDL total	Comprehensive evaluation	74	0.129	−0.103 to 0.347	0.274
CT total	Theory score	77	0.003	−0.221 to 0.227	0.980
CT open-mindedness	Weekly assignment score	77	0.244	0.021 to 0.444	0.032
CT inquisitiveness	Case score	77	0.292	0.073 to 0.484	0.010

Pearson correlation coefficients (*r*), 95% confidence intervals, and two-sided *p* values are reported. Domain-level analyses were exploratory; none of the 55 domain-level Pearson associations remained significant after Benjamini–Hochberg FDR adjustment.

### 3.4 Multivariable linear regression

For multivariable modeling, theory score was specified as the dependent variable (*n* = 73) with self−directed learning total and critical thinking total as predictors and age, sex, and final surgical nursing course score as covariates. After adjustment, critical thinking total was not independently associated with theory score (*β* = 0.014, 95% CI − 0.103 to 0.131; *p* = 0.817), and self-directed learning total was also not statistically significant (*β* = −0.110, 95% CI − 0.226 to 0.007; *p* = 0.065). None of the covariates were independently associated with theory score (all *p* > 0.05), and the overall model was not statistically significant (*p* = 0.211) (Model A) (Table 4).

**Table 4:** Multivariable linear regression model for theory examination score.

Predictor	*β*	SE	95% CI (lower)	95% CI (upper)	*P*	VIF
Self-directed learning total (per 1-point)	−0.110	0.059	−0.226	0.007	0.065	1.229
Critical thinking total (per 1-point)	0.014	0.059	−0.103	0.131	0.817	1.223
Final surgical nursing course score (per 1-point)	0.109	0.152	−0.194	0.412	0.475	1.003
Age (per 1 year)	−2.406	1.433	−5.266	0.454	0.098	1.123
Sex (male = 1, female = 0)	−5.793	3.898	−13.574	1.988	0.142	1.115

Multivariable linear regression with theory examination score as the dependent variable. β denotes unstandardized coefficients. Models were adjusted for age, sex, and final surgical nursing course score; 95% CIs and two-sided *p* values are reported. Adjusted for Age, Sex, and Final Surgical Nursing Course Score. VIF denotes variance inflation factor. Overall model statistics: *R*^2^ = 0.099, adjusted *R*^2^ = 0.032, *F*(5,67) = 1.471, *p* = 0.211.

When the comprehensive end−of−rotation evaluation score was modeled (*n* = 73), neither self−directed learning total (*β* = 0.046, 95% CI − 0.080 to 0.171; *p* = 0.469) nor critical thinking total (*β* = 0.058, 95% CI − 0.068 to 0.184; *p* = 0.361) showed an independent association. The overall model was not statistically significant (*F*-test *p* = 0.566) (Model B) (Table 5).

**Table 5:** Multivariable linear regression model for comprehensive end-of-rotation evaluation score.

Predictor	*β*	SE	95% CI (lower)	95% CI (upper)	*P*	VIF
Self-directed learning total (per 1-point)	0.046	0.063	−0.080	0.171	0.469	1.229
Critical thinking total (per 1-point)	0.058	0.063	−0.068	0.184	0.361	1.223
Final surgical nursing course score (per 1-point)	0.082	0.163	−0.244	0.408	0.617	1.003
Age (per 1 year)	1.313	1.541	−1.763	4.389	0.397	1.123
Sex (male = 1, female = 0)	5.884	4.193	−2.486	14.254	0.165	1.115

Multivariable linear regression with comprehensive end-of-rotation evaluation score as the dependent variable. β denotes unstandardized coefficients. Models were adjusted for age, sex, and final surgical nursing course score; 95% CIs and two-sided *p* values are reported. VIF denotes variance inflation factor. Overall model statistics: *R*^2^ = 0.055, adjusted *R*^2^ = −0.015, *F*(5,67) = 0.783, *p* = 0.566.

### 3.5 Sensitivity and robustness analyses

CT total score was not significantly associated with theory examination score using either Pearson correlation (r = 0.003, *p* = 0.980) or Spearman rank correlation (*ρ* = 0.019, *p* = 0.870) (Supplementary Tables S2, S3). In standardized regression, CT total score was not significantly associated with theory examination score (standardized *β* = 0.030, *p* = 0.817) (Table 6). In the additional sensitivity model using the same complete-case sample (*n* = 73) but excluding the final surgical nursing course score, the CT–theory association remained non-significant (*β* = 0.015, 95% CI − 0.102 to 0.131; *p* = 0.801). Given the restricted age distribution, the theory-score model was also refitted without age; the CT–theory association remained non-significant (*β* = 0.010, 95% CI − 0.109 to 0.128; *p* = 0.872). Regression diagnostics showed low VIF values and non-significant Breusch–Pagan and Ramsey RESET tests, while maximum Cook’s distances were 0.217 and 0.137. Shapiro–Wilk tests indicated departures from residual normality in both models (Supplementary Table S4).

**Table 6:** Sensitivity and robustness analyses.

Analysis	*n*	Effect size	*P*	Adjustment/notes
Sensitivity regression excluding age: CT total	73	*β* = 0.010 (95% CI − 0.109 to 0.128)	0.872	Adjusted for SDL total, sex, and final surgical nursing course score; same complete-case sample as the theory-score model
SDL total vs. comprehensive evaluation (adjusted partial *r*)	73	r_partial = 0.137	0.257	Adjusted for age, sex, and final surgical nursing course score
CT total vs. comprehensive evaluation (adjusted partial *r*)	75	r_partial = 0.161	0.178	Adjusted for age, sex, and final surgical nursing course score
SDL total vs. case score (adjusted partial *r*)	73	r_partial = 0.005	0.966	Adjusted for age, sex, and final surgical nursing course score
CT total vs. theory score (Spearman correlation)	77	*ρ* = 0.019	0.870	Rank-based sensitivity analysis
Standardized regression (theory score model): CT total	73	*β*_std = 0.030	0.817	Theory score and continuous predictors were standardized (z-scores); sex was retained as a binary variable
Sensitivity regression excluding final surgical nursing course score: CT total	73	*β* = 0.015 (95% CI − 0.102 to 0.131)	0.801	Adjusted for SDL total, age, and sex; same complete-case sample as the theory-score model

Partial correlations (r_partial) were adjusted for the specified covariates. Spearman correlations (ρ) and standardized regression coefficients (*β*_std) were used for sensitivity analyses. Regression sensitivity models used the same complete-case sample as the theory-score model. All *p* values are two-sided.

## 4 Discussion

In this cohort of nursing interns rotating through urology, we evaluated whether self-directed learning and critical thinking dispositions showed outcome-specific relationships across routinely recorded assessment components. The results indicate that associations differed by outcome type: neither critical thinking disposition nor self-directed learning total scores were independently associated with theory examination performance after covariate adjustment, and comprehensive instructor evaluations were also not independently associated with either ability construct. However, exploratory domain-level analyses showed nominal positive associations of CT open-mindedness with weekly assignment performance and CT inquisitiveness with case presentation performance, both of which were also observed in the Spearman analyses. Neither association, nor any other domain-level association, remained statistically significant after FDR adjustment. Accordingly, the domain-level findings should be regarded as hypothesis-generating rather than confirmatory. Given the single-specialty, single-institution setting and the exploratory nature of the domain-level analyses, these observations should be considered preliminary and context-specific. The observed pattern is consistent with previous work suggesting that associations between learner attributes and performance may vary according to assessment type and format (12), and supports further evaluation across different assessment components and settings.

After covariate adjustment, critical thinking disposition was not independently associated with the written theory examination outcome. This null association remained after exclusion of the final surgical nursing course score and in the Spearman analysis. Therefore, the present data do not support an inverse relationship between critical thinking disposition and theory examination performance. One possible interpretation is that critical thinking disposition and conventional written examinations capture partially different aspects of learning. Importantly, the CTDI-CV measures a dispositional tendency toward critical thinking rather than demonstrated critical-thinking competence or clinical reasoning performance. The theory examination used in this study primarily assessed knowledge retrieval and application and was therefore not expected to serve as a direct measure of critical-thinking competence. Biggs’ model of learning approaches provides a theoretical basis for expecting associations between learner attributes and performance to vary across assessment formats (16). Empirically, Fero et al. (12) demonstrated this method-dependency directly in nursing students: CCTDI disposition scores were not associated with performance in paper-based vignettes, but were significantly associated with high-fidelity simulation performance, an assessment modality that more authentically samples clinical reasoning processes. Wangensteen et al. (17) also examined critical thinking disposition in relation to research utilization among newly graduated nurses, supporting the possibility that the relevance of critical thinking disposition varies with the outcome assessed, although differences across study settings preclude direct comparison.

Viewed through an assessment-design lens, the results point to a possible construct-coverage gap: routine grading instruments may not be sufficiently sensitive to the self-reported learning attributes and critical thinking dispositions measured by self-report SDL and CT scales (4). The selected internship outcomes were not designed as direct measures of demonstrated critical-thinking competence. Weekly assignments primarily reflected process-oriented learning engagement and self-regulation, whereas case presentations required clinical information organization, prioritization, and structured communication and therefore may theoretically engage some reasoning-related processes without constituting a validated measure of clinical reasoning or critical-thinking competence. Internships blend knowledge testing with professional behaviors and task completion, so a single global rating integrates professionalism, teamwork, and overall impression across the rotation and may not align closely with the constructs assessed by self-report SDL and CT disposition measures (2, 4). This limitation is well-recognized in the workplace-based assessment literature. Govaerts et al. (18) demonstrated that instructor ratings in clinical settings are heavily influenced by rater cognition, idiosyncratic performance theories, and contextual cues, and may reflect the rater’s subjective impression of the trainee rather than the learner attributes assessed by self-report instruments. Williams et al. (19) further identified that cognitive, social, and environmental factors introduce systematic bias into clinical performance ratings, reducing their ability to discriminate between learners who differ primarily in cognitive dispositions. Taken together, these findings suggest that the non-significant relationship between SDL/CT disposition measures and the comprehensive instructor rating in this study may partly reflect measurement limitations of the evaluation instrument itself, rather than a true absence of any association.

The absence of a significant independent association between SDL and either outcome in adjusted models also warrants discussion. Although SDL has been consistently linked to positive learning outcomes and clinical competence development in some studies (5, 11), others have found that this relationship is substantially mediated by contextual factors in the clinical learning environment (7). In the current study, interns rotating through a single specialty ward were subject to the same supervision structure, workload, and available learning resources, which may have constrained the variance in SDL-relevant behaviors and thereby attenuated any detectable association with assessment scores. Furthermore, routine examination formats and global instructor ratings may not adequately capture the incremental contribution of SDL-specific behaviors such as self-initiated information seeking, proactive goal setting, and independent reflection (11) activities that are more likely to manifest in competency assessments that include a process or portfolio dimension. Restricted variability in some performance indicators may also have reduced the ability to detect associations; this was particularly evident for practical skills and case presentation scores, which were concentrated within relatively narrow ranges. The small effect sizes for the comprehensive instructor rating may additionally reflect ceiling effects: the mean score was 92.48 out of 100, consistent with the well-documented leniency tendency in summative workplace-based assessments, where social dynamics between supervisors and trainees and the desire to support interns often compress the upper range of ratings and reduce their discriminative power (18, 20).

This work has several strengths. First, it links validated learner ability measures to multiple routinely collected internship outcomes, enabling outcome-specific interpretation rather than reliance on a single summary score, an approach that is increasingly recommended in nursing education research (3, 4). Second, the analyses incorporated covariate adjustment for age, sex, and prior academic performance, and included sensitivity checks using Spearman rank correlations and standardized regression. The use of multiple performance indicators also reduced reliance on any single instructor-rated outcome, which may be influenced by cognitive, social, and environmental sources of rating bias (19). Third, because the study used rotation records generated during usual educational practice rather than a specially designed research instrument, the findings have direct relevance to existing clinical education systems and can inform practical decisions about assessment design and learner support without requiring additional data infrastructure. Importantly, the predominance of null and non-robust associations is itself informative, as it suggests that routinely used internship assessments may not closely align with the learner attributes captured by SDL and critical thinking disposition measures. This observation helps define a potential construct-coverage gap and supports the development or selection of assessment approaches that more directly capture self-regulated learning, clinical reasoning, and other targeted competencies.

Several limitations merit consideration. The study was conducted within a single specialty rotation at one institution, and the findings may not generalize to other clinical settings, specialty contexts, or broader student populations. The observational cross-sectional design precludes causal inference, and covariate adjustment was limited to age, sex, and final surgical nursing course score. Age varied only from 20 to 23 years, and the sample was predominantly female; therefore, these demographic variables provided limited information for adjustment in this cohort, and their individual regression coefficients should not be interpreted as stable estimates of age or sex-related effects. Although exclusion of age in sensitivity analysis did not materially alter the CT–theory estimate, unmeasured educational and clinical factors—including broader measures of prior academic achievement, prior clinical exposure, supervisor characteristics, workload, and the clinical learning environment—were not available in the parent dataset. Their omission may have resulted in residual confounding and omitted-variable bias, potentially shifting the estimated associations between SDL/CT disposition and internship performance in either direction. Formal documentation regarding theory-examination blueprinting, content coverage, item-level difficulty, and psychometric consistency across cohorts was not available. Similarly, detailed scoring anchors, formal records of rater training, and inter-rater reliability or other formal validity evidence for the comprehensive instructor evaluation were unavailable. The comprehensive instructor evaluation may be susceptible to leniency bias and ceiling effects, which may have attenuated its associations with self-report SDL and CT disposition measures. The relatively narrow distributions of practical skills and case presentation scores may likewise have reduced their ability to discriminate between interns and attenuated observable correlations. Accordingly, these routine educational assessments should be interpreted as internship performance indicators rather than validated direct measures of specific competencies. The self-report format of both the SDL and CT instruments introduces the possibility of social desirability bias, and although 80 interns were included overall, the effective sample sizes for several analyses were approximately 73–77. The confidence intervals around the principal null associations indicate that the study provides reasonable precision for excluding large associations but does not exclude small, and for some outcomes modest, relationships of potential educational relevance. For example, the CT total–theory score correlation was close to zero but had a 95% CI of −0.221 to 0.227, whereas the SDL total–comprehensive evaluation correlation had a 95% CI of −0.103 to 0.347. Thus, the non-significant findings should be interpreted as an absence of clearly demonstrated associations in this cohort rather than definitive evidence of no association. In addition, the small amount of missing data reduced the effective sample size in some analyses; although no imputation was performed, and no separate imputation-based sensitivity analysis was undertaken because missingness was limited, reduced precision or bias related to missingness cannot be completely excluded. The exploratory domain-level analyses comprised 55 comparisons, creating a substantial opportunity for chance findings. Although several associations had unadjusted *p* < 0.05, none remained statistically significant after FDR adjustment. These domain-level findings should therefore be considered hypothesis-generating and require independent confirmation.

## 5 Conclusion

In this urology nursing rotation cohort, self-directed learning and critical thinking disposition showed non-uniform associations across different internship performance indicators. After covariate adjustment, neither critical thinking disposition nor self-directed learning ability was independently associated with theory examination performance. Routine comprehensive instructor evaluations were not significantly associated with either self-directed learning or critical thinking disposition in adjusted analyses. The null association between critical thinking disposition and theory examination performance was also consistent in the rank-based sensitivity analysis and additional sensitivity regression models. These findings suggest that routinely used internship assessment components may not fully capture the learner attributes represented by SDL and critical thinking disposition measures, highlighting the need for more construct-aligned assessment approaches. Overall, these findings should be considered preliminary and context-specific and require replication across other clinical rotations and institutions before broader conclusions are drawn regarding the alignment between learner attributes and internship assessment components.

## Data Availability

The original contributions presented in the study are included in the article/Supplementary material, further inquiries can be directed to the corresponding author.
