# Supplementary material for: Associations of self−directed learning ability and critical thinking disposition with internship performance indicators in nursing interns
Source: Front Public Health. 2026 Oct 16;14:1945090. doi: 10.3389/fpubh.2026.1945090 (PMC13719615; doi:10.3389/fpubh.2026.1945090)
Supplement: Supplementary file 1 [file Table_1.DOCX]

Supplementary Table S1. Availability of Study Variables

| Variable | Available, n | Missing, n | Missing, % |
| --- | --- | --- | --- |
| Age | 80 | 0 | 0 |
| Final surgical nursing course score | 78 | 2 | 2.5 |
| SDL total score | 80 | 0 | 0 |
| CT total score | 80 | 0 | 0 |
| Theory examination score | 77 | 3 | 3.8 |
| Practical skills average score | 77 | 3 | 3.8 |
| Weekly assignment average score | 80 | 0 | 0 |
| Case score | 80 | 0 | 0 |
| Comprehensive end-of-rotation evaluation score | 80 | 0 | 0 |

Notes: Available n reflects variable-level availability; effective matched sample sizes for correlation analyses are reported in Supplementary Tables S2 and S3. Correlation analyses were conducted using matched questionnaire and performance records (n=74 for SDL analyses and n=77 for CT analyses).

Supplementary Table S2. Pearson Correlations of SDL/CT Scores with Internship Performance Indicators

| Ability measure | Theory examination | Practical skills | Weekly assignment | Case score | Comprehensive evaluation |
| --- | --- | --- | --- | --- | --- |
| SDL total | −0.164 (0.162) | 0.018 (0.881) | 0.012 (0.922) | 0.034 (0.775) | 0.129 (0.274) |
| SDL motivation | −0.261 (0.025) | 0.002 (0.989) | −0.091 (0.440) | 0.075 (0.523) | 0.144 (0.220) |
| SDL self-management | −0.116 (0.324) | 0.016 (0.892) | 0.116 (0.326) | 0.033 (0.778) | 0.097 (0.413) |
| SDL collaboration | 0.084 (0.477) | 0.074 (0.533) | 0.030 (0.799) | −0.066 (0.574) | 0.130 (0.268) |
| SDL information literacy | −0.241 (0.039) | −0.015 (0.896) | −0.061 (0.603) | 0.044 (0.707) | 0.094 (0.424) |
| CT total | 0.003 (0.980) | 0.068 (0.556) | −0.010 (0.928) | 0.216 (0.060) | 0.120 (0.297) |
| CT truth-seeking | −0.082 (0.476) | 0.125 (0.277) | −0.080 (0.490) | 0.181 (0.115) | 0.003 (0.982) |
| CT open-mindedness | 0.077 (0.508) | 0.205 (0.074) | 0.244 (0.032) | 0.082 (0.476) | −0.004 (0.976) |
| CT analyticity | 0.112 (0.331) | 0.116 (0.315) | 0.040 (0.730) | 0.146 (0.204) | 0.112 (0.332) |
| CT systematicity | 0.078 (0.501) | 0.026 (0.824) | 0.080 (0.489) | 0.169 (0.143) | 0.158 (0.171) |
| CT confidence in reasoning | −0.217 (0.058) | −0.186 (0.105) | −0.062 (0.591) | −0.287 (0.011) | −0.150 (0.193) |
| CT inquisitiveness | 0.024 (0.838) | −0.022 (0.847) | −0.103 (0.370) | 0.292 (0.010) | 0.097 (0.403) |
| CT cognitive maturity | −0.070 (0.545) | −0.088 (0.448) | −0.178 (0.122) | 0.069 (0.552) | 0.138 (0.230) |

Notes: Values are Pearson correlation coefficients (r) with two-sided P values in parentheses. Effective sample sizes were n=74 for SDL analyses and n=77 for CT analyses. Domain-level analyses were exploratory.

Supplementary Table S3. Spearman Correlations of SDL/CT Scores with Internship Performance Indicators

| Ability measure | Theory examination | Practical skills | Weekly assignment | Case score | Comprehensive evaluation |
| --- | --- | --- | --- | --- | --- |
| SDL total | −0.102 (0.386) | −0.005 (0.963) | 0.057 (0.629) | 0.012 (0.921) | 0.126 (0.286) |
| SDL motivation | −0.214 (0.067) | −0.006 (0.962) | −0.037 (0.752) | 0.072 (0.542) | 0.153 (0.192) |
| SDL self-management | −0.037 (0.756) | −0.035 (0.766) | 0.173 (0.141) | 0.029 (0.809) | 0.110 (0.351) |
| SDL collaboration | 0.130 (0.270) | 0.051 (0.664) | 0.072 (0.542) | −0.064 (0.586) | 0.120 (0.310) |
| SDL information literacy | −0.217 (0.063) | −0.040 (0.735) | −0.019 (0.873) | 0.042 (0.722) | 0.080 (0.498) |
| CT total | 0.019 (0.870) | −0.015 (0.899) | 0.060 (0.607) | 0.183 (0.111) | 0.053 (0.646) |
| CT truth-seeking | −0.081 (0.482) | 0.052 (0.651) | −0.084 (0.467) | 0.125 (0.279) | −0.023 (0.843) |
| CT open-mindedness | 0.009 (0.937) | 0.164 (0.153) | 0.250 (0.028) | 0.098 (0.398) | −0.112 (0.332) |
| CT analyticity | 0.141 (0.220) | 0.074 (0.521) | 0.138 (0.230) | 0.097 (0.402) | 0.090 (0.434) |
| CT systematicity | 0.046 (0.691) | −0.025 (0.831) | 0.108 (0.350) | 0.091 (0.429) | 0.128 (0.266) |
| CT confidence in reasoning | −0.131 (0.257) | −0.117 (0.310) | −0.043 (0.708) | −0.283 (0.013) | −0.130 (0.261) |
| CT inquisitiveness | −0.009 (0.935) | −0.054 (0.642) | −0.024 (0.836) | 0.328 (0.004) | 0.067 (0.563) |
| CT cognitive maturity | −0.058 (0.618) | −0.071 (0.538) | −0.015 (0.899) | 0.106 (0.358) | 0.079 (0.495) |

Notes: Values are Spearman rank correlation coefficients (ρ) with two-sided P values in parentheses. Effective sample sizes were n=74 for SDL analyses and n=77 for CT analyses. These analyses were used as sensitivity analyses; domain-level findings were exploratory.

Supplementary Table S4. Regression Diagnostic Statistics

| Diagnostic | Theory examination model | Comprehensive evaluation model |
| --- | --- | --- |
| VIF range | 1.003–1.229 | 1.003–1.229 |
| Durbin–Watson statistic | 1.814 | 1.476 |
| Breusch–Pagan test, P | 0.672 | 0.563 |
| Shapiro–Wilk W | 0.940 | 0.901 |
| Shapiro–Wilk test, P | 0.002 | <0.001 |
| Maximum Cook’s distance | 0.217 | 0.137 |
| Ramsey RESET test, P | 0.170 | 0.242 |

Notes: VIF, variance inflation factor. Breusch–Pagan and Ramsey RESET tests were used to assess homoscedasticity and model specification, respectively. Cook’s distance was used to assess influential observations. Residual normality was evaluated using the Shapiro–Wilk test.
